# The relationship between knowledge, attitude, and practice of plasma donation with age and gender as moderators: a cross-sectional survey

**DOI:** 10.1097/MS9.0000000000000121

**Published:** 2023-03-16

**Authors:** Kashif Tousif, Sajeel Saeed, Sidra Hamid, Tehseen Haider, Jawad Basit, Abdul Rauf Khalid, Muaz Ali, Rubaid Azhar Dhillon, Mohammad Ebad Ur Rehman, Muhammad Farhan

**Affiliations:** a Department of Medicine; b Department of Physiology; c Department of Surgery, Rawalpindi Medical University, Rawalpindi, Punjab; d Department of Medicine, Riphah International University, Islamabad, Islamabad Capital Territory, Pakistan

**Keywords:** attitude, COVID-19, knowledge, plasma, practice

## Abstract

Convalescent plasma therapy has gained worldwide notoriety since its inception as a form of soul treatment for terminally ill patients. This study investigates the link between knowledge, attitude, and practice of plasma donation while simultaneously probing the moderative role of age and gender in this context.

**Method:**

A cross-sectional study was undertaken in Rawalpindi, Pakistan, on COVID-19-recovered patients (coronavirus disease 2019). In all, 383 persons were chosen using simple random sampling. A prestructured questionnaire was first validated and then used as a tool for data collection. jMetrik version 4.1.1 and SPSS version 26 were used to enter and analyze the data. Reliability analysis, hierarchal regression, and logistic regression analysis were applied.

**Results:**

A total of 85.1% and 58.2% of 383 individuals had a favorable attitude and sufficient knowledge toward plasma donation, respectively. Plasma donation was observed in 109 (28.5%) of the individuals. Plasma donation practice was shown to be strongly related to plasma donation attitude [adjusted odds ratio (AOR)=4.48; *P*<0.05] and knowledge (AOR=3.78; *P*<0.001). Females who had more plasma donation knowledge and attitude tend to donate more compared to males. However, no interactional effect of gender×knowledge and attitude, and age×knowledge and attitude was found with plasma donation practice.

**Conclusion:**

Plasma donation was uncommon, even though the majority of individuals had a good mindset and were well-informed. Fear of getting a health problem was linked to the decreased practice.

Highlights

- 85.1% and 58.2% of 383 individuals had good attitudes and good knowledge toward plasma donation, respectively.
- Plasma donation was seen in 109 (28.5%) of the individuals.
- Plasma donation practice was shown to be strongly related to plasma donation attitude [adjusted odds ratio (AOR)=4.48; *P*<0.05] and knowledge (AOR=3.78; *P*<0.001).

## Introduction

Severe acute respiratory syndrome coronavirus 2, a novel coronavirus, has spread swiftly over the world, triggering a global pandemic. There are now 234 million individuals around the globe who are afflicted by this disease^1^. The clinical presentation of severe acute respiratory syndrome, which has become the main cause of death in the affected population, is diverse^2^–^4^. It does not have a predilection for a particular age, as it can affect any age group, whether children or adults. Studies have shown that more than 70% of adults are usually mildly symptomatic, while only a few experience a severe life-threatening infection, whereas children most often have a less severe form of the disease, with the majority of them having mild symptoms like fever (53.9%) and cough (39.9%)^5^,^6^. Apart from being a global health catastrophe, the pandemic has aggravated into a political, social, and economic downfall and chaos^7^. Pakistan is one of the most gravely affected countries with 1.2 million cases and 27,000 deaths to date^8^.

Numerous treatment strategies have been attempted, but none are reliable or acceptable. Certain diseases for which no specific therapeutic approach exists have historically been treated using convalescent plasma (CP) therapy, a kind of passive immunotherapy^9^. The use of CP has shown beneficial effects on critically ill patients, decreasing hospital stay and mortality rates^10^,^11^. According to Cheng et al.^12^, who examined about 2000 severe acute respiratory syndrome patients who received CP died at a lower rate than those who did not. Plasma therapy is changing and underpinning the treatment of disease in specific therapeutic contexts.

In the wake of this pandemic, several marketing campaigns were held about coronavirus disease 2019 (COVID-19) infection and its subsequent treatment with CP therapy to increase community knowledge regarding plasma donation. Higher knowledge can become a driving factor for a higher attitude and practice of plasma donation. However, there is a widespread reluctance among the COVID-19-recovered individuals to donate plasma as they feel they will lose their hard-earned immunity. Due to these contradictory circumstances, there is a pressing need to examine plasma donation knowledge, attitude, and practice in light of the current epidemic. Though many studies have been conducted to gauge the efficacy of CP therapy^12^–^14^, the amount of medical literature addressing the knowledge and attitude of plasma donation is relatively scarce. The lack of concrete research on this topic limits the understanding of the knowledge and attitude toward plasma donation.

As a result of this research, recovered patients will be more receptive to donating plasma to others in need, as they will have a better appreciation for the critical nature of plasma donation knowledge and attitude. We examined the frequency of plasma donation across different professions and educational levels as part of our study.

## Methods

### Study design

To assess plasma donation’s knowledge, attitude and practice, a cross-sectional study was conducted among COVID-19-recovered patients from January to September 2021 in Rawalpindi, Pakistan. The participants involved in the study were COVID-19 survivors located only within Rawalpindi. All of these participants tested negative 2 or more weeks prior. Participants who could not donate because of medical issues were excluded.

### Ethical approval

Before the initiation of the study, the Institutional Review Board (IRB) of Islamic International Medical College Riphah, Rawalpindi, Pakistan, issued ethical approval (ERC/21/#291) for conducting the research. The data-collecting method was carried out in compliance with national and institutional ethical guidelines, as well as the most recent version of the Helsinki Declaration. Individual consent was acquired from each participant. Data anonymity and confidentiality were preserved. The manuscript follows the STROCSS (Strengthening The Reporting Of Cohort Studies in Surgery) guidelines for reporting a cross-sectional study^15^.

### Sample size

Data were obtained from 1800 people through a convenient nonprobability sampling technique, with 1494 forms filled out and meeting our inclusion criteria, resulting in an 83% correct response rate. With the use of the WHO sample size calculator, a sample size of 383 was determined, with a 0.05 as the margin of error (*d*), 95% confidence level, population size (*N*) of 1800, and nonresponse rate of 17%. The expected proportion (*P*) of knowledge and attitude toward plasma donation was taken as 0.5 in the population. The formula for sample size calculation is as follows:


$$n=\frac{z_{1-\frac{\alpha }{2}}^{2}P(1-P)N}{d^{2}(N-1)+z_{1-\frac{\alpha }{2}}^{2}P(1-P)}.$$

So out of 1494 forms, the lottery sampling technique (simple random sampling technique) was then employed for data selection.

### Data collection procedure

COVID-19’s mechanism of transmission through human contact necessitated the use of an online platform to collect responses. A self-administered questionnaire, after reviewing previously validated questionnaires from similar research that had been published^16^–^20^, was devised and it was then used as a tool for data collection. The initial questionnaire consisted of 11 items of knowledge and 12 items of attitude. The questionnaire was sent to five subject experts, which included a public health expert, two physicians, and two medical educationists. Data from expert opinions were analyzed using Lawshe’s method to see if the scale instrument could stably represent the defined universe of the content it was supposed to measure or its specific fields and if it was quantitatively and qualitatively efficient enough to measure the behavior (feature) that was supposed to be measured. The content validity ratio and content validity index values of the subtests, as well as the whole scale instrument, were determined to be 1.00 using Lawshe’s approach. As a result, it was concluded that each item should be included in the scale, and the test’s content validity was confirmed. Four questions from the knowledge and two questions from the attitude portion were then subsequently removed based on their comments. Before moving forward with the survey, all participants were required to express their desire to volunteer for involvement, and filling out the form was taken as their permission. Before completing the survey, a brief overview of the study questionnaire, research objectives, and instructions were provided. The questionnaire consisted of 29 questions, including 6 questions regarding demographic details, there are 7 questions about knowledge, 10 questions about attitude, and 6 questions about plasma donation practice. Before disseminating the survey, a pilot study was conducted with 20 participants to ensure clarity and compliance. Following this, the questionnaire was updated accordingly, and these responses were excluded from the final data.

### Reliability analysis

The questionnaire’s reliability was assessed using Cronbach’s *α*. The internal consistency of the questionnaire was 0.74. A Cronbach’s *α* value for the knowledge scale was 0.67 for seven questions, whereas for the attitude scale was 0.68 for 10 questions. For the whole scale, reliability coefficients were determined, along with a 95% confidence range, and standard error values for the reliability coefficient are shown in Table 1.

**Table 1:** Reliability analysis of the ‘knowledge and attitude of plasma donation’ scale.

Method	Estimate	Confidence interval lower bound	Confidence interval upper bound	SEM
Knowledge scale				
Gutman’s L2	0.6943	0.6451	0.7388	0.9029
Coefficient *α*	0.6677	0.6142	0.7161	0.9413
Raju’s β	0.6677	0.6142	0.7161	0.9413
Feldt–Brennan	0.6890	0.6390	0.7344	0.9106
Feldt–Gilmer	0.6985	0.6501	0.7425	0.8965
Attitude scale				
Gutman’s L2	0.7084	0.6628	0.7500	1.0473
Coefficient *α*	0.6781	0.6278	0.7241	1.1002
Raju’s β	0.6781	0.6278	0.7241	1.1002
Feldt–Brennan	0.6962	0.6487	0.7396	1.0689
Feldt–Gilmer	0.7055	0.6596	0.7476	1.0523

### Data entry and analysis

SPSS version 26 and jMetrik version 4.1.1 were used to collect and analyze data. Frequencies and central tendencies were used for descriptive analysis. To examine the relationship between the dependent and explanatory variables, binomial and multinomial logistic regression models were employed. All *P* values under 0.05 were deemed significant. Every correct answer got a knowledge and attitude score of ‘1,’ while every incorrect answer received a knowledge and attitude score of ‘0.’

## Results

### Demographic details

This research included 383 people. The median age was 33 years (interquartile range=20). The bulk of the participants was aged 21–40 (62.7%). Males made up 207 (54%) of the total subjects, while females made up 176 (46%) of the total subjects. When it came to marital status, 237 (61.9%) individuals were married. In terms of occupation, 63 (16.4%) were government employees, 99 (25.8%) were private workers, and 89 (23.2%) participants were students. In terms of education, 175 individuals (45.7%) were postgraduates. The most common blood type among the study’s participants was O+, which accounted for 21.1% of respondents, while 7.6% of all subjects were unsure of their blood group.

### Knowledge of plasma donation

The concept of knowledge was classified into two categories: ‘High Knowledge’ and ‘Low Knowledge.’ This was done by using the median as a cut point value, which in this instance was 4, and establishing a criterion of sufficient knowledge as a total score of 4/7 (58.2%) or above. However, 223 (58.2%) of participants had sufficient knowledge. The mean knowledge score of the subjects was 56.65±23.32 SD. A total of 353 (92.2%) participants were familiar with the term ‘plasma’ and 215 (56.1%) participants were aware of the minimum age for donating. While 282 (73.6%) of participants were aware of their entitlement to voluntary plasma donation, the majority lacked sufficient information about the time gap between consecutive donations, the amount of plasma collected during donation, and the weight criterion for plasma donation (Table 2).

**Table 2:** COVID-19-recovered patients’ knowledge of plasma donation.

Questions	Responses	Frequencies, *n* (%)	Correct answer, *n* (%)	Incorrect answer, *n* (%)
Do you have any idea what plasma is?	Yes	353 (92.2)	353 (92.2)	30 (7.8)
	No	30 (7.8)		
Do you know how much weight you need to donate plasma?	Yes	56 (14.6)	56 (14.6)	327 (85.4)
	No	327 (85.4)		
Do you know the minimum age for plasma donation?	Yes	215 (56.1)	215 (56.1)	168 (43.9)
	No	168 (43.9)		
Do you know the interval between two consecutive donations?	Yes	139 (36.3)	139 (36.3)	244 (63.7)
	No	244 (63.7)		
Do you know the volume of plasma taken during the donation?	Yes	115 (30)	115 (30)	268 (70)
	No	268 (70)		
	Yes	24 (6.3)	359 (93.7)	24 (6.3)
Do you think donating plasma adversely affects health?	No	359 (93.7)		
Are you aware of your right to donate plasma voluntarily?	Yes	282 (73.6)	282 (73.6)	101 (26.4)
	No	101 (26.4)		

COVID-19, coronavirus disease 2019.

### Attitude toward plasma donation

The mean attitude score was 80.94 with a standard deviation of 19.39. A positive attitude was defined as having a total score of 7/10 (70%) or above. 326 (85.1%) individuals had a favorable opinion regarding the donation of plasma, whereas 57 (14.9%) expressed a negative view. 306 (79.9%) of the participants expressed an interest in plasma donation. The selling of plasma was deemed immoral by 370 (96.6%) participants. Over 75% of participants indicated an interest in promoting and teaching others about plasma donation and its advantages. Plasma therapy was deemed a successful treatment for COVID-19 by 338 (88.3%) participants. 290 (75.7%) individuals saw plasma giving as a moral obligation. 90.3% of 346 participants thought that plasma donation would not affect their immunity (Table 3).

**Table 3:** COVID-19-recovered patients’ attitudes regarding plasma donation.

			Attitude	
Questions	Responses	Frequencies, *n* (%)	Positive, *n* (%)	Negative, *n* (%)
Is plasma donation something you’d consider doing?	Yes	306 (79.9)	306 (79.9)	77 (20.1)
	No	77 (20.1)		
Do you think the selling of plasma is ethical?	Yes	13 (3.4)	370 (96.4)	13 (3.4)
	No	370 (96.4)		
Will you encourage people for plasma donation?	Yes	298 (77.8)	298 (77.8)	85 (22.2)
	No	85 (22.2)		
Will you take part in educating COVID-19 patients regarding plasma donation?	Yes	290 (75.7)	290 (75.7)	93 (24.3)
	No	93 (24.3)		
Do you think plasma therapy is an effective treatment for COVID-19 patients?	Yes	338 (88.3)	338 (88.3)	45 (11.7)
	No	45 (11.7)		
Will you donate plasma to an unknown person?	Yes	241 (62.9)	241 (62.9)	142 (37.1)
	No	142 (37.1)		
Do you believe that donating plasma is a moral obligation?	Yes	290 (75.7)	290 (75.7)	93 (24.3)
	No	93 (24.3)		
Do you believe that giving plasma reduces immunity?	Yes	37 (9.7)	346 (90.3)	37 (9.7)
	No	346 (90.3)		
What will you do to a person who is selling plasma?	Convince	295 (77)	295 (77)	88 (23)
	Do nothing	88 (23)		
Do you think donating plasma spreads infection?	Yes	57 (14.9)	326 (85.1)	57 (14.9)
	No	326 (85.1)		

COVID-19, coronavirus disease 2019.

To determine the sample adequacy for the knowledge scale, the Kaiser–Meyer–Olkin (KMO test) value was 0.726 and the Bartlett *χ* ^2^ value was 479.24 (df=55, *P*=0.05). To determine the sampling adequacy of the attitude scale, the Kaiser–Meyer–Olkin (KMO test) value was 0.727 and the *χ* ^2^ value of Bartlett was 582.665 (df=45, *P*=0.05).

### Practice of plasma donation

In practice, individuals were classified as practicing plasma donors if they had at least one history of plasma donation. Plasma donation was reported to be practiced by 109 (28.5%) individuals, with 87 (22.7%) individuals having given just one time and 22 (5.7%) respondents having contributed several times. Of them, 91 (83.48%) plasma donors contributed voluntarily, 5 (4.6%) sold plasma, and 13 (11.92%) donated as a replacement. 303 (79.1%) of 383 participants expressed a future readiness to donate plasma. 28.5% of participants were donors, with 274 (71.5%) being nondonors. Health concerns (25.55%), not being asked (24.45%), and fear of contracting HIV (14.96%) were all significant reasons for not giving, as were family resistance, fear of needles, and concerns about cleanliness.

### Factors associated with knowledge of plasma donation

Males had more knowledge [adjusted odds ratio (AOR)=1.37; 95% CI: 0.86–2.18] than females. In comparison to the 61–80 age group, those aged 1–20 (AOR=0.35; 95% CI: 0.004–2.80), 21–40 (AOR=1.02; 95% CI: 0.18–5.79), and 41–60 (AOR=0.54; 95% CI: 0.09–3.21) were less informed. Students (AOR=8.00; 95% CI: 1.80–35.53) had more knowledge than professionals (AOR=8.00; 95% CI: 1.80–35.53). Unmarried individuals (AOR=1.06; 95% CI: 0.54–2.07) had more knowledge than married individuals. Plasma donation practice (AOR=3.78; 95% CI: 2.19–6.54) was significantly related with plasma donation knowledge (AOR=3.78; 95% CI: 2.19–6.54) (see Table 4).

**Table 4:** Association of demographic factors with knowledge of plasma donation.

	Knowledge			
Variables	Adequate	Inadequate	COR (95% CI)	AOR (95% CI)
Gender				
Male	133	74	1.71 (1.14–2.58)*	1.37 (0.86–2.18)
Female	90	86	1	1
Age groups				
1–20	15	11	2.27 (0.63–8.14)	0.35 (0.04–2.80)
21–40	152	88	2.87 (1.01–8.19)*	0.77 (0.13–4.58)
41–60	50	41	1.63 (0.55–4.83)	0.54 (0.09–3.21)
61–80	6	10	1	1
Occupation				
Student	64	25	4.24 (2.29–7.83)**	8.00 (1.80–35.53)*
Private employee	61	38	2.66 (1.50–4.71)**	1.36 (0.44–4.23)
Government employee	42	21	3.31 (1.71–6.42)**	1.56 (0.46–5.26)
Housewife	38	63	1	1
Other	18	13	2.29 (1.01–5.20)*	2.92 (0.71–11.94)
Marital status				
Married	128	109	1	1
Unmarried	95	51	1.58 (0.03–2.42)*	1.06 (0.54–2.07)
Education				
Matric	2	8	1	1
Inter	20	21	3.81 (0.72–20.15)	4.12 (0.68–24.87)
Postgraduate	115	60	7.66 (1.58–37.24)*	7.75 (1.39–43.15)*
Blood group known				
Known	211	143	2.09 (0.97–4.50)	1.95 (0.85–4.50)
Unknown	12	17	1	1
Practice of plasma donation				
Yes	88	21	4.31 (2.53–7.34)**	3.78 (2.19–6.54)**
No	135	139	1	1

AOR, adjusted odds ratio; COR, crude odds ratio.

* Significance with *P*<0.05.

** Significance with *P*<0.001.

### Factors associated with attitude toward plasma donation

Males had a lower probability of having a positive attitude (AOR=0.78; 95% CI: 0.42–1.45) than females. When compared to married individuals, unmarried people (AOR=1.17; 95% CI: 0.47–2.94) showed a more positive attitude regarding plasma donation. Plasma donation practice (AOR=4.48; 95% CI: 1.71–11.72) was shown to be substantially related to attitudes regarding plasma donation (Table 5).

**Table 5:** Association of demographic variables with attitude of plasma donation.

	Attitude			
Variables	Good	Bad	COR (95% CI)	AOR (95% CI)
Gender				
Female	150	26	1	1
Male	176	31	0.98 (0.55–1.73)	0.78 (0.42–1.45)
Age groups				
1–20	23	3	3.48 (0.70–17.28)	0.28 (0.02–4.94)
21–40	208	32	2.95 (0.96–9.06)	0.39 (0.05–3.39)
41–60	84	17	2.24 (0.69–7.29)	0.42 (0.05–3.50)
61–80	11	5	1	1
Occupation				
Housewife	82	19	1	1
Student	82	7	2.71 (1.08–6.80)*	2.90 (0.30–27.47)
Government employee	52	11	1.09 (0.48–2.48)	1.52 (0.28–8.14)
Private employee	86	13	1.53 (0.71–3.30)	1.93 (0.38–9.59)
Other	24	7	0.79 (0.29–2.11)	1.36 (0.21–8.97)
Marital status				
Unmarried	131	15	1.88 (1.00–3.53)*	1.17 (0.47–2.94)
Married	195	42	1	1
Education				
Postgraduate	146	29	2.15 (0.52–8.83)	2.02 (0.43–9.47)
Graduate	140	17	3.52 (0.83–14.94)	3.49 (0.75–16.21)
Inter	33	8	1.76 (0.37–8.39)	1.59 (0.29–8.50)
Matric	7	3	1	1
Blood group known				
Unknown	20	9	1	1
Known	306	48	2.87 (1.23–6.66)*	2.24 (0.92–5.47)
Practice of plasma donation				
Yes	104	5	4.87 (1.89–12.55)**	4.48 (1.71–11.72)*
No	222	52	1	1

AOR, adjusted odds ratio; COR, crude odds ratio.

* Significance with *P*<0.05.

** Significance with *P*<0.001.

The interrelationship between knowledge score, attitude score, and plasma donation practice was investigated using hierarchal regression analysis while controlling for age and gender. In step 1, age was negatively associated with plasma donation practice indicating that the population in the young age group was more involved in the donation of plasma. Gender was also negatively associated with the practice of plasma donation, which indicated that males were more involved in plasma donation practice compared to females. In step 2, the knowledge and attitude score showed a positive association with plasma donation practice. This indicated that females who had more knowledge and attitude values were more involved in the practice of plasma donation. The covariate variables explained 13% of the variance in plasma donation practice. In step 3, the interaction effect of knowledge score×age, knowledge score×gender, attitude score×age, and attitude score×gender was nonsignificant, showing that the strength of association between knowledge and attitude scores and plasma donation practice did not differ significantly between age and gender. In sum, the model explained 13% of the total variance in plasma donation practice (Table 6).

**Table 6:** Summary of the hierarchical regression analysis for variables predicting the practice of plasma donation.

	Plasma donation				
	*B*	SEB	*β*	*F*	Adjusted *R* ^2^
Step 1					
Constant	0.49	0.09			
Age	−0.01	0.01	−0.08		
Gender	−0.07	0.05	−0.08	2.51	0.08
Step 2					
Constant	−1.07	0.14			
Age	−5.429E−05	0.01	−0.01		
Gender	−0.03	0.04	−0.04		
Knowledge score	0.01	0.01	0.35*		
Attitude score	0.01	0.01	0.03	15.05	0.13
Step 3					
Constant	−6.45	0.42			
Age	0.01	0.01	0.11		
Gender	0.22	0.19	0.24		
Knowledge score	0.01	0.01	0.54*		
Attitude score	0.01	0.01	−0.21		
Knowledge score×age	−5.953E−05	0.01	−0.13		
Knowledge score×gender	−0.01	0.01	−0.09		
Attitude score×age	−7.618E−06	0.01	−0.02		
Attitude score×gender	−0.01	0.01	−0.27	7.85	0.13

Gender: male=1, female=2.

* Significance in hierarchical regression analysis with *P*<0.05.

** Significance in hierarchical regression analysis with *P*<0.001.

## Discussion

For several life-threatening illnesses, plasma treatment is successful, including hemophilia, Ebola virus sickness, and certain immunodeficiency diseases^21^,^22^. Currently, the whole world is in the jaws of a pandemic, that is COVID-19, as new treatments are continually being tested in addition to the presence of several effective vaccines. In such a state, people must know different aspects of the disease, its prevention, and treatment. According to a study on healthcare professionals in Pakistan, the majority of doctors have adequate knowledge regarding COVID-19 transmission and its prevention^23^. COVID-19 individuals with life-threatening illnesses may benefit greatly from plasma therapy. Plasma therapy has helped tens of thousands of individuals who are dependent on ventilators all over the globe^24^. As a consequence, plasma donation should be given consideration. It will be simpler to fight COVID-19 if more individuals give plasma. Additionally, it has a variety of positive effects on the health of donors^25^. The public’s perception of plasma donation is important in light of all these considerations.

According to the findings of this research, blood donation knowledge and attitude levels follow a similar pattern. Research in North India found that 74.4% of participants had knowledge level^26^; however, this survey’s findings revealed just 223 (58.2%) had sufficient knowledge. To say that this epidemic has had such an effect on people’s awareness of plasma treatment and plasma donation is an understatement. This may be due to plasma therapy’s widespread use on social media and many public awareness efforts. It was shown that individuals with an intermediate, graduate, or postgraduate degree had a better understanding of plasma donation. Education’s beneficial impact on people’s knowledge may be shown in higher levels of knowledge linked with advanced degrees. Awareness campaigns on plasma donation may be made more effective if they are encouraged and held more often.

According to the findings, 326 (85.1%) individuals had a favorable opinion about plasma donation. The results are consistent with public perceptions about blood donation. Positive attitude levels were lower than those found in an Ethiopian study (94.5%)^27^ but higher than those found in a similar poll in Basrah, Iraq (68.7%)^28^. A potential reason for this kind of response is because the participants have had comparable difficulties in their own lives and may sympathize with the patients. Again, the favorable attitude of recent college graduates and postgraduates demonstrates how educational achievement influences how an individual thinks about certain subjects.

Despite a favorable attitude and enough knowledge, only 28.5% of individuals surveyed had previously donated plasma. According to the study, this practice is less prevalent in Ethiopia (12.5%) than it is in Tamil Nadu (31%)^29^. Another study conducted in Sudan found that about 60.6% of plasma donors were males^30^. Men have an edge when it comes to plasma donation, owing to the challenges women face throughout the process. Due to a lack of knowledge and societal pressure, women are discouraged from donating plasma. Plasma donation was considerably more prevalent among individuals between the ages of 21 and 40 than in any other age group. Other studies have shown a higher prevalence in the year-old age group, which this one verifies^17^. This increased frequency may be explained by the fact that adults have a better degree of knowledge and are more socially involved than children and adolescents. Individuals who were aware of their blood type reported being happier and having more knowledge than those who were unaware.

Our results indicate that health concerns accounted for 25.55% of the reasons individuals did not give blood, even when no one explicitly asked it, and fear of acquiring HIV accounted for 24.45 and 14.96%, respectively. Additionally, there was a lack of support from friends and family, worries about hygiene, a needle fear, and concerns about the possible health implications. Nondonors cited similar reasons in other studies^31^ as well. Additionally, education and initiatives aimed at raising awareness for emergency patients about the need for CP treatment may help address the issue.

There has been no prior study done on plasma donation, as far as we know. The strength of this study is that it was done at the community level enabling us to find an association between these outcome variables and certain factors in the community that could affect plasma donation.

The majority of the population supported volunteerism, seeing it as a moral duty. Donors, governments, organizations, hospitals, and patients should all collaborate to establish a coordinating triangle to guarantee the safety and ethical treatment of donations. People’s apprehension regarding plasma donation may be alleviated via public awareness campaigns that address typical worries about the process.

Numerous restrictions apply to our research. We cannot establish a causal relationship between the outcome and the explanatory variables since this is a cross-sectional study. Due to the limited sample size, the results cannot be generalized to the whole country. We cannot rule out reporting bias since donors did not provide proof of plasma donation; we can only assume they are donors based on their questionnaire responses.

## Conclusion

Despite having an excellent mentality and sufficient information, the majority of the participants had poor plasma donation practices in their daily routines. The lack of participation was caused by people being afraid of becoming sick, such as HIV or having a health problem. Education level was strongly linked to plasma donating knowledge. People who knew their blood type were more prepared and more willing to donate plasma. To contemplate practicing plasma donation, a person’s attitude and understanding regarding plasma treatment must be favorable.

## Ethical approval

The Institutional Review Board (IRB) of Islamic International Medical College Riphah, Rawalpindi, Pakistan, issued the ethical approval (ERC/21/#291) for conducting the research.

## Patient consent

Prior consent was taken from every participant before filling up the google form.

## Sources of funding

No funding was required for this study.

## Conflicts of interest disclosure

The authors declared no potential conflicts of interest.

## Author contribution

K.T. and T.H.: conceptualized and had major contributions in devising the methodology and writing and editing the manuscript. S.S.: conceptualized and had major contributions in devising the methodology, analyzing the data, and writing and editing the manuscript. S.H.: supervised the investigation and had major contributions in devising the methodology and writing and editing the manuscript. J.B., A.R.K., M.A., and R.A.D.: collected the data virtually and had major contributions in writing and editing the manuscript. M.E.U.R. and M.F.: critical review and approval of the final version.

## Research registration unique identifying number (UIN)

1. Name of the registry: not applicable.
2. Unique identifying number or registration ID: not applicable.
3. Hyperlink to your specific registration (must be publicly accessible and will be checked): not applicable.

## Guarantor

Sajeel Saeed: Department of Medicine, Rawalpindi Medical University, Block E Satellite Town, Rawalpindi, Pakistan.

## Provenance and peer review

Not commissioned, externally peer-reviewed.
